# Review of quantitative and functional lung imaging evidence of vaping-related lung injury

**DOI:** 10.3389/fmed.2024.1285361

**Published:** 2024-01-24

**Authors:** Joseph J. Hofmann, Victoria C. Poulos, Jiahai Zhou, Maksym Sharma, Grace Parraga, Marrissa J. McIntosh

**Affiliations:** ^1^Robarts Research Institute, London, ON, Canada; ^2^Department of Medical Biophysics, London, ON, Canada; ^3^Department of Medical Imaging, Western University, London, ON, Canada

**Keywords:** e-cigarettes, MRI, CT, PET, EVALI

## Abstract

**Introduction:**

The pulmonary effects of e-cigarette use (or vaping) became a healthcare concern in 2019, following the rapid increase of e-cigarette-related or vaping-associated lung injury (EVALI) in young people, which resulted in the critical care admission of thousands of teenagers and young adults. Pulmonary functional imaging is well-positioned to provide information about the acute and chronic effects of vaping. We generated a systematic review to retrieve relevant imaging studies that describe the acute and chronic imaging findings that underly vaping-related lung structure-function abnormalities.

**Methods:**

A systematic review was undertaken on June 13th, 2023 using PubMed to search for published manuscripts using the following criteria: [(“Vaping” OR “e-cigarette” OR “EVALI”) AND (“MRI” OR “CT” OR “Imaging”)]. We included only studies involving human participants, vaping/e-cigarette use, and MRI, CT and/or PET.

**Results:**

The search identified 445 manuscripts, of which 110 (668 unique participants) specifically mentioned MRI, PET or CT imaging in cases or retrospective case series of patients who vaped. This included 105 manuscripts specific to CT (626 participants), three manuscripts which mainly used MRI (23 participants), and two manuscripts which described PET findings (20 participants). Most studies were conducted in North America (*n* = 90), with the remaining studies conducted in Europe (*n* = 15), Asia (*n* = 4) and South America (*n* = 1). The vast majority of publications described case studies (*n* = 93) and a few described larger retrospective or prospective studies (*n* = 17). In e-cigarette users and patients with EVALI, key CT findings included ground-glass opacities, consolidations and subpleural sparing, MRI revealed abnormal ventilation, perfusion and ventilation/perfusion matching, while PET showed evidence of pulmonary inflammation.

**Discussion and conclusion:**

Pulmonary structural and functional imaging abnormalities were common in patients with EVALI and in e-cigarette users with or without respiratory symptoms, which suggests that functional MRI may be helpful in the investigation of the pulmonary health effects associated with e-cigarette use.

## Introduction

In the summer of 2019, public health authorities in the US issued a public health alert and launched an investigation which resulted in the reporting of a large cluster of hospitalizations in young electronic cigarette (e-cigarette) users (1). The outbreak, first identified in July 2019 (2), stemmed from the hospital admission of five previously healthy teens who were recent e-cigarette users, which was then reported by the Centers for Disease Control (CDC) in the US (3). Because of this concerted public health approach, nearly 3,000 cases of idiopathic acute lung injury were reported in the fall of 2019, a few months prior to the beginning of the coronavirus disease 2019 (COVID-19) pandemic. Serious, life-threatening lung injury requiring critical care and mechanical ventilation and, in some cases, extracorporeal membrane oxygenation (ECMO), was reported primarily in previously healthy adolescents, which lead to the deaths of at least 68 adolescents and young adults (4), the youngest of whom was 13 years of age (5). Lung injury related to e-cigarette use was contemporaneously termed EC-related or vaping product use-associated lung injury (EVALI) (6). While the number and intensity of vaping related hospitalizations diminished during the pandemic years, many unanswered questions remain about the impact of vaping on lung structure and function in combustible cigarette users and in people who had never smoked combustible cigarettes prior. Such questions included: “what was the exact compound and pathophysiologic mechanisms responsible for the e-cigarette-related acute lung injury in 2019?”; “what are the acute and chronic effects of vaping on pulmonary health?”; “with the public health alert now passed, do unreported intensive care hospital admissions still occur in young adults and adolescents?; “how do the different e-liquid components impact the lungs?”; and, finally, “how does the apparent lung damage that stems from vaping or e-cigarette use directly compare to the acute and long term effects of combustible cigarette use?”

Unfortunately, there are very few studies designed to answer these questions, and even fewer studies which provide high quality evidence based on randomized, controlled prospective research designs. This systematic review was undertaken to uncover and summarize the pulmonary imaging evidence already published and explore some of these unanswered questions.

## Historical context and technical developments

### Historical development of vaping-related devices

The first e-cigarette was developed and patented in 1930 (U.S. Patent No. 1775947A) for individual use and a similar device was patented in 1963 (U.S. Patent No. 3200819A); neither of these devices were manufactured for commercialization. In 2003, a Chinese pharmacist, Hon Lik, initially developed the e-cigarette as an alternative to combustible cigarettes. It was commercialized in 2004 in Canada and China and was on the market in Europe and the United States in 2006 (7).

**Figure 1:**
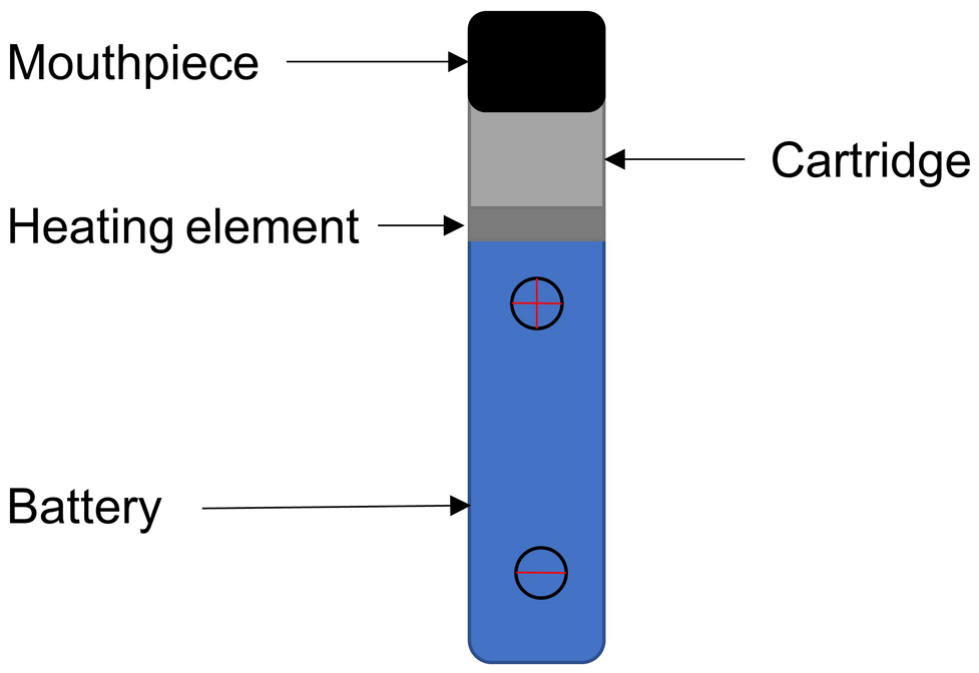
Typical commercial electronic cigarette or vape device.

As shown in Figure 1, modern e-cigarette devices consist of a battery, mouthpiece, heating element, liquid solution reservoir and disposable cartridge or pod. The battery powers a heating element which, when applied to the liquid solution, rapidly increases the temperature enabling the transition of the e-liquid to a gaseous or aerosol state, which is subsequently inhaled via a mouthpiece into the lungs.

### E-cigarette versus combustible cigarette risk

The long-term use of combustible cigarettes can cause various cardiopulmonary health risks such as chronic obstructive pulmonary disease, pulmonary hypertension and cancer. Despite some national advisory committees suggesting that the relative health risks of e-cigarette use are reduced as compared to combustible cigarettes (8), very few head-to-head comparison studies have been completed. Previous investigations suggest that e-cigarette use, either alone or in combination with combustible cigarettes, is associated with reduced overall health, breathing difficulties, and cardiovascular abnormalities (9, 10). Several systematic reviews have been conducted to examine the impact of e-cigarette use on respiratory illness (11) and human health (12); here, we summarize pulmonary imaging findings in e-cigarette users.

### Composition of vaping e-liquids

**Figure 2:**
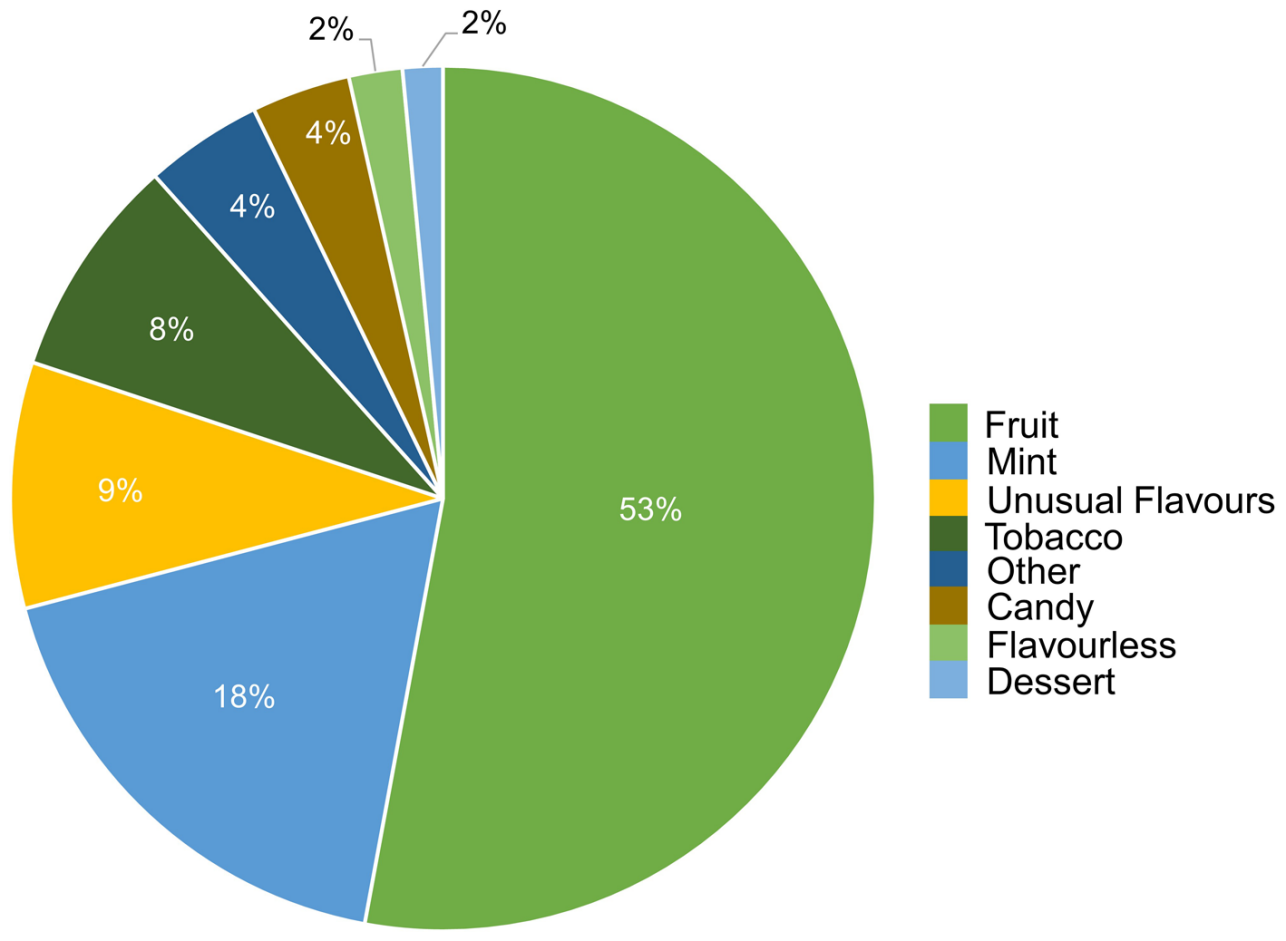
Distribution of vape flavors by preference. Figure was created using data from Canadian Tobacco and Nicotine Survey (CTNS) (14).

e-liquids contain flavors, solvent carriers and active ingredients such as tetrahydrocannabinol (THC) or nicotine. The solvent carriers typically consist of propylene glycol, ethylene glycol, glycerol, tobacco-specific nitrosamines, volatile organic compounds, phenolic compounds, tobacco alkaloids, aldehydes, free radicals, reactive oxygen species, furans and metals (i.e., nickel, lead, chromium). Flavoring substances include menthol, ethyl maltol and diacetyl which are present in the most popular flavors of e-cigarettes (13), as shown in Figure 2.

#### Flavors and excipients

Table 1 lists common excipients and flavors present in e-liquids. Active ingredients and concentrated flavoring compounds are dissolved in inactive excipients, which are then delivered to users as an aerosol.

**Table 1:** Common excipients and flavors in e-liquids.

Chemical	Molecular structure	Function	Impact on lung health
**Excipients**			
Diacetyl	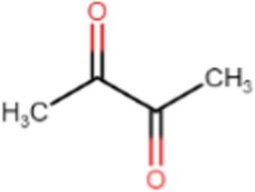	Buttery flavor	Associated with bronchiolitis obliterans (15)
Propylene glycol	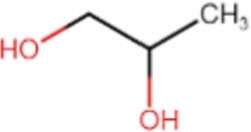	Emulsifies ingredients to increase solidity	Generates pulmonary irritants and carcinogenic carbonyl compounds (16)
Vegetable glycerin (glycerol/glycerin)	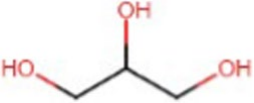	Delivery vehicle	Increases mucin expression primary airway epithelia (17)
Vitamin E acetate (alpha-tocopherol acetate)	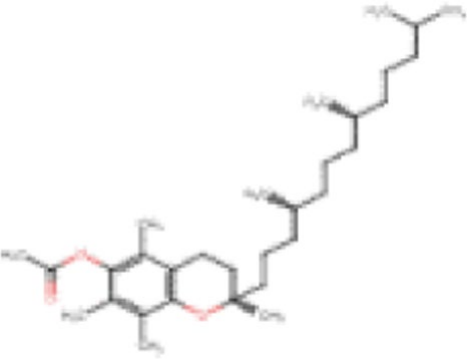	Thicken or dilute e-liquids with THC derivatives	Alters surfactant expansion and compression cycles (18) Linked to EVALI (19)
**Flavors**			
Cinnamaldehyde	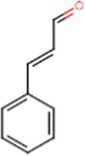	Cinnamon	Suppression of macrophage phagocytosis (20) Alters cell morphology and motility (21) DNA strand breakage due to oxidative burst (21)
Vanillin	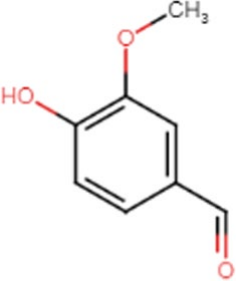	Vanilla	Airway epithelial cell metabolic disruption (22) Metabolic effect amino acids, fatty acids, lipids and mitochondrial function (22)
Ethyl vanillin	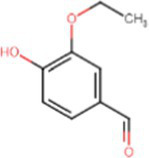	Vanilla	Decreased neutrophil oxidative burst (23)
Ethyl maltol	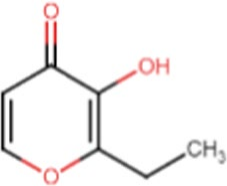	Caramel	Co-exposure with copper causes epithelial cell apoptosis and DNA damage (24)
Menthol	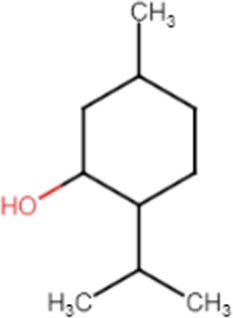	Mint	Decreases cell proliferation (25) Increased oxidative stress (25) Damages respiratory epithelium (25)
Benzaldehyde	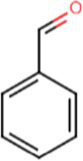	Cherry-almond	Attenuates oxidative burst capacity of neutrophil (21)
Linalool	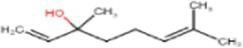	Floral, sweet	Increases cytotoxicity (21)

Molecular structure representations were generated with Marvin SJ (26). EVALI, e-cigarette-related or vaping-associated lung injury.

There are more than 7,000 vape liquid flavors and over 450 brands available on the market (27). Though many flavorings fall under the “generally recognized as safe” (GRAS) provision by the US Food and Drug Administration (FDA), the GRAS status only applies to the use of such flavorings in ingested foods and not for inhaled products (28). In fact, at least 65 individual flavoring ingredients in flavored e-liquids were observed to cause toxicity in the respiratory track by inducing cytotoxicity, generating reactive oxygen species and impairing clearance mechanisms (21). Furthermore, cinnamaldehyde, vanillin, menthol, ethyl vanillin, benzaldehyde, ethyl maltol and linalool were present in the flavors that caused the most toxicity in *in vitro* studies (21). Unfortunately, the variety of products available as well as the ongoing modifications of e-cigarette and vaping devices makes it difficult to comprehensively evaluate the biological risk of vaping and specific e-liquids (29).

As early as 2000 (15, 30), the excipient diacetyl, which provides a buttery flavor in e-liquids (31), was associated with the development of bronchiolitis obliterans or “popcorn lung” in workers at a microwave popcorn plant; inflammation and fibrosis in the lung can result in the partial or complete obstruction of the peripheral airway lumen, thus leading to bronchiolitis obliterans.

Previous work has demonstrated that propylene glycol, which acts as an emulsifier for active ingredients in e-liquids (32), may also damage peripheral airways by harming epithelial cells and reducing cell proliferation; e-cigarette users with underlying chronic obstructive pulmonary disease (COPD) were more susceptible to small airway epithelial damage than those without (33).

Vegetable glycerin serves as a delivery vehicle for the active contents in e-liquids (34). Vegetable glycerin e-cigarette aerosols disturb the human nasal cystic fibrosis transmembrane conductance regulator, resulting in consequent mucus hyperconcentration and potentially harming the airway by inducing inflammation and ion channel dysfunction (34).

Vitamin E acetate is commonly used as a thickening agent in vaping products that contain cannabis derivatives (31). The inhalation of vitamin E acetate may lead to impaired pulmonary function (35–37). Furthermore, when heated, vitamin E acetate thermally degrades into ketene, alkene and benzene, all of which may contribute to epithelial lung injury (18). Additional studies observed that vitamin E acetate alters surfactant expansion and compression cycles, potentially compromising surfactant function (38). With compromised lung surfactant, the alveolar surface tension would increase and may cause an inflammatory cascade in lung tissue. The CDC previously observed vitamin E acetate in the bronchoalveolar lavage fluid of 94% of EVALI patients (19, 39), pointing to an association between vitamin E acetate and the development of EVALI.

#### Active ingredients

Commonly used active ingredients in e-liquids include THC and nicotine. THC is the psychoactive compound in cannabis and may be added to e-liquids (21). While THC can be degraded to toxins such as methacrolein and benzene when heated in vapes (18), other compounds in THC-based e-liquids may also have negative effects on respiratory health. THC-based e-liquids differ from nicotine-based liquids because THC (a highly hydrophobic chemical) requires a hydrophobic emulsifier to be incorporated into the vape liquid (18). Thus, vitamin E acetate, described in the previous section, is commonly used in THC-based e-liquids. While the vast majority of EVALI cases (80%–86%) are associated with vaping THC products, the overall health risks of cannabis vaping is largely unknown (18, 29, 39, 40).

e-cigarettes are marketed as a safe alternative to traditional combustible cigarettes as they do not contain carcinogenic incomplete combustion byproducts nor tobacco nitrosamines (41). However, previous investigations have demonstrated an association between nicotine and vaping-induced COPD pathologies (42, 43), such as emphysema, as well as an increased risk of lung cancer among nicotine e-cigarette users (44). Although vaping does not require tobacco or combustion, stimulant nicotine in e-liquids may still cause DNA damage (45) and may mediate tumor growth by promoting the self-renewal of stem-like cells in tumor initiation and metastasis (46).

## Pathophysiology of vaping-induced lung injury

### Symptoms

The majority of EVALI patients experience respiratory symptoms, including cough, chest pain, shortness of breath and hemoptysis (2, 39, 47), in addition to gastrointestinal symptoms, such as abdominal pain, nausea, vomiting and diarrhea; gastrointestinal symptoms may precede respiratory symptoms in some patients (47). Additional common symptoms include unexplained weight loss, headache and fatigue (2), and, more generally, EVALI patients are frequently admitted with tachycardia, tachypnea, fever and hypoxemia (39). Because lung biopsies not commonly obtained in EVALI patients, the pathological drivers of symptoms and disease progression remain poorly understood.

### The respiratory system

The respiratory system consists of two main parts: the upper and lower respiratory tract. The upper tract includes the nose, nasal cavity, throat and larynx. This tract is responsible for bringing in air from outside the body, through the nose and mouth.

**Figure 3:**
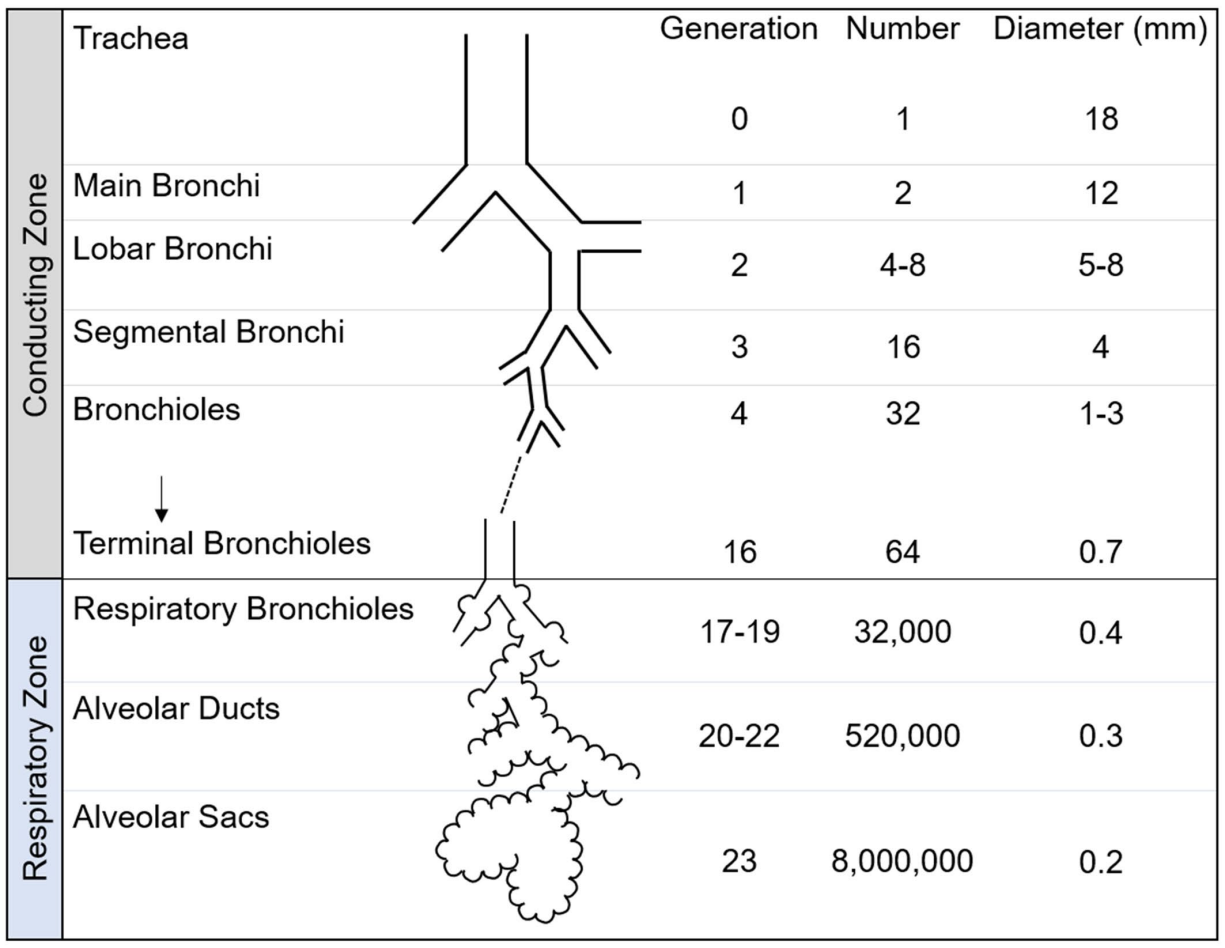
Schematic of airway tree conducting and respiratory zone. The human airway tree consists of the conducting zone and respiratory zone, with corresponding generation, number and diameter shown. Adapted from Nunn’s Applied Respiratory Physiology, 8th edition.

As shown in Figure 3, the lower respiratory tract is divided into two zones, known as the conducting and respiratory zones. These zones encompass a total of 23 generations of airways. The conducting zone, which includes airway generations 0–16, is responsible for guiding inhaled air towards the alveoli and humidifying it. The conducting zone begins with the trachea which then bifurcates across 16 generations of airways to the terminal bronchioles, which conclude the conducting zone. As the airways progress into further generations, their diameter gradually decreases. The airways within the conducting zone consist of thick walls of mucosa, smooth muscle and cartilage and are lined with cilia to remove dust and foreign particles from the lung.

The respiratory zone is responsible for facilitating gas exchange and contains airway generations 17–23. The respiratory zone contains respiratory bronchioles, alveolar ducts and alveolar sacs. Within the lungs, there are numerous microscopic sacs known as alveoli, which begin to bud along the walls of the respiratory bronchioles and become increasingly abundant with each subsequent airway generation. These alveoli are surrounded by a network of capillaries, whose primary function is to facilitate the exchange of inhaled oxygen with carbon dioxide. The alveolar sacs complete the respiratory zone and the airway tree.

### Pathophysiology

**Figure 4:**
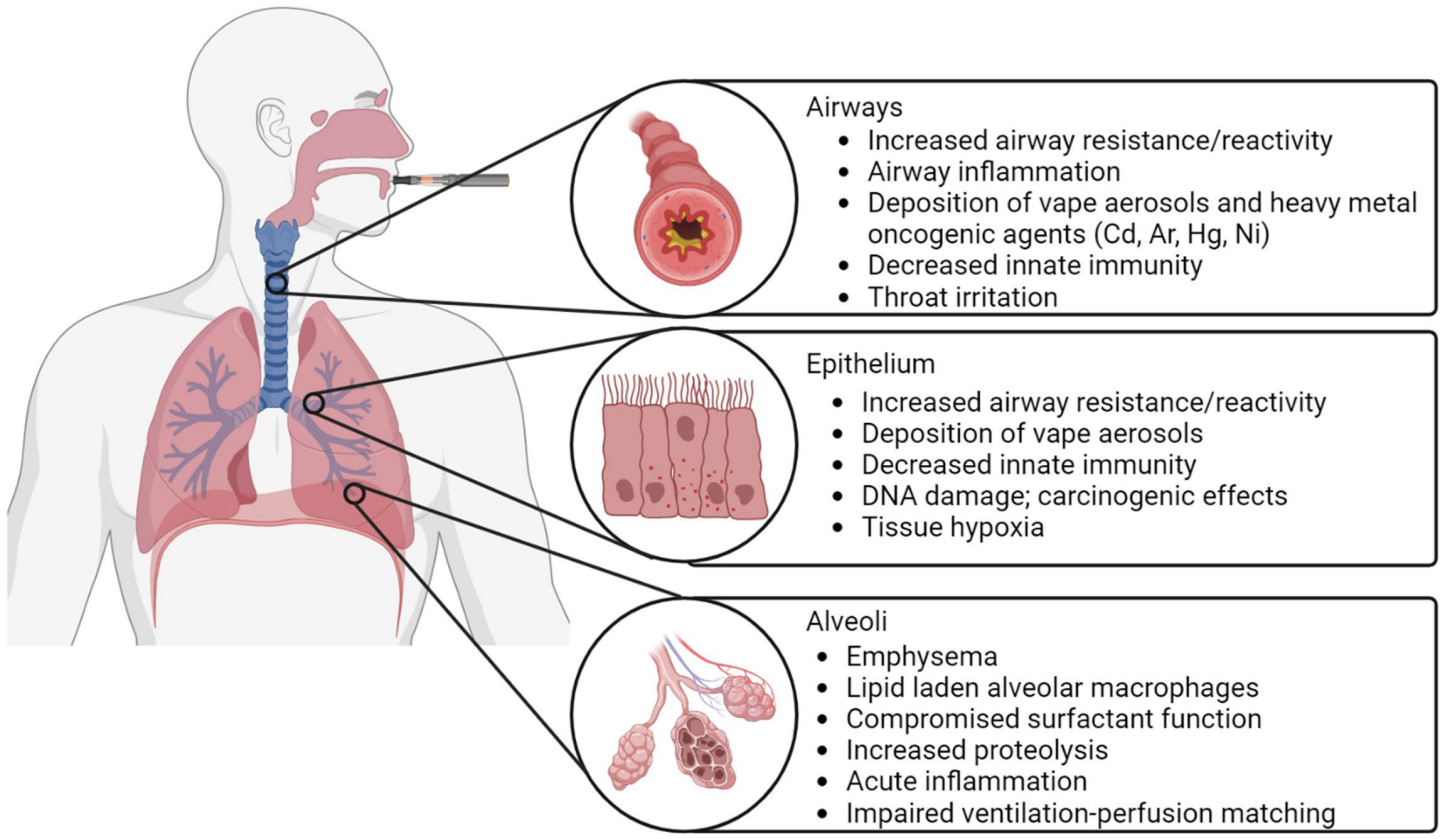
Potential mechanisms of pulmonary injury associated with vaping.

Potential mechanisms of vaping-related lung injury are provided in schematic in Figure 4. When aerosolized e-liquids are inhaled, the particles come in direct contact with the entire respiratory system. Consequently, chemicals in e-cigarette aerosols, such as menthol and ethyl maltol may lead to inflammation, which is thought to be the dominant cause or contribution to vaping-related lung injury (18, 31, 48–50). It has also been postulated that the pro-inflammatory effects caused by vape aerosols are partially mediated by reactive oxygen species (ROS) (27), which may lead to cellular apoptosis through ROS-mediated autophagy (51); this cellular death pathway is also mechanistically attributed to emphysema (27). In addition, pulmonary inflammation and fibrosis may result in bronchiolitis obliterans, which is characterized by hypertrophy of the bronchiolar smooth muscle, peribronchiolar inflammatory infiltrates, mucus accumulation in the bronchiolar lumen and bronchiolar scarring (52). This scarring is irreversible and bronchiolitis obliterans has no known cure. While healthy patients may achieve complete improvement, the pulmonary health of most patients progressively worsens and some may even require mechanical ventilation or lung transplants in severe cases (53).

Heavy metals have been detected in the vape aerosols produced from pod-type vapes (27), including chromium, nickel, copper, zinc, cadmium, tin, manganese and lead (54). The metallic components of e-cigarette devices, such as the filaments and coils, are comprised of such heavy metals and can degrade when exposed to oxidized acidic e-liquids (54). While metal exposure is a risk factor for multiple pulmonary diseases including respiratory inflammation, asthma, COPD and respiratory cancer (55–57), the health effects of metal exposure in e-cigarette users is still largely unknown (58).

Patients with EVALI or vaping-related lung injury consistently present with lipid laden alveolar macrophages or foam cells in the lung or in bronchoalveolar lavage fluid (59). These macrophages serve as the main phagocytes in the innate immune system, clearing the airspaces of potentially harmful particles (48). Recent work has observed lipid laden alveolar macrophages in the bronchoalveolar lavage fluid of 80% of EVALI patients, demonstrating its potential as a biomarker of vaping-related lung injury. Nevertheless, the presence of lipid laden alveolar macrophages is not unique to vaping, which has been reported in a number of other pulmonary conditions (18). Thus, they are a non-specific marker of vape product use, and do not provide a direct prognosis of vaping-related lung injury (60).

### Knowledge and health care gaps

There are currently many unanswered questions associated with vaping. An unexhausted list of these questions includes: “what are the longitudinal effects of vaping on pulmonary health?”; “what is the safety profile of e-cigarette use relative combustible cigarettes?”; “what are the effects of each e-liquid component on respiratory health?”; and “what role can pulmonary imaging play in the diagnosis and monitoring of vaping-related lung injury?”

This review is designed to explore some of these unanswered questions in the context of pulmonary imaging.

### Research questions

We aim to investigate the current understanding of vaping in literature, the conditions associated with it, and the common imaging modalities used to evaluate vaping-related lung injury, specifically magnetic resonance imaging (MRI), computed tomography (CT) and positron emission tomography (PET).

## Methods

This review was conducted according to the preferred reporting items for systematic reviews and meta-analyses (PRISMA). We used PubMed to search for manuscripts related to vaping/e-cigarette use or EVALI and pulmonary imaging on June 13, 2023 using the terms [(“Vaping” OR “e-cigarette” OR “EVALI”) AND (“MRI” OR “CT” OR “Imaging”)].

### Inclusion and exclusion criteria

The inclusion and exclusion criteria are presented in Table 2. The inclusion criteria were: (a) imaging modalities: strictly MRI, CT and PET; (b) bodily systems scanned; strictly structures in the respiratory system: such as lungs, respiratory epithelium, and alveoli; (c) test subjects: living human subjects who had previously used e-cigarettes/vapes. Exclusion criteria included: (1) any types of non-electric cigarette such as conventional cigarettes, joints, cigars, and hookahs; (2) any articles that were reviews or not directly treating a single/group of patients.

**Table 2:** Inclusion and exclusion criteria for systematic review.

Parameter	Inclusion	Exclusion
Imaging modalities	MRI, CT, PET	Other
Body system	Respiratory	Non-respiratory
Test subjects	Human	Non-human
Type of vape	Electronic cigarettes	Combustible cigarettes (primary use)
Type of article	Case study, case series, retrospective studies, prospective studies	Review articles, others

MRI, magnetic resonance imaging; CT, computed tomography; PET, positron emission tomography.

### Selection process and data collection

Four reviewers (JH, VP, JZ, and GP) independently screened the abstracts of the retrieved reports to evaluate whether they met the predetermined inclusion/exclusion criteria. A full text review of the remaining studies was performed to further evaluate the inclusion eligibility of the reports and any discrepancies were presented and discussed between the four reviewers to ensure a consensus was made regarding the inclusion of each report.

### Risk of bias assessment

Three reviewers (JH, VP, and JZ) independently appraised the methodological qualities of the included studies in accordance with two risk of bias tools, detailed further in Supplementary Figure S1. The JBI critical appraisal checklist for case reports was deemed most appropriate for one report [Eddy et al. (61)] while the Cochran risk of bias in non-randomized studies-of exposure tool (ROBINS-E) was used for the remaining studies. The JBI tool assesses bias arising from: the patient’s demographics (D1), history (D2) and clinical condition on presentation (D3), the diagnostic tests or assessment methods (D4), the interventions or treatments used (D5), the post-intervention clinical condition (D6), the adverse effects on unanticipated events (D7), and the takeaway lessons (D8). The ROBINS-E tool assesses bias arising from: confounds (D1), measurement of the exposure (D2), selection of the participants in the study (D3), post-exposure interventions (D4), missing data (D5), measurement of the outcome (D6), and selection of the reported result (D7). The level of risk judgement for each domain was categorized into four categories: high risk, some concerns, low risk, or no information.Synthesis methods

The criteria used to determine which studies were eligible for each synthesis was that, for each patient, the study must report age, gender, location, and history of e-cigarette or vape use. We used a table to separate patient characteristics such as age, type of e-device vaped and symptoms. No data conversions were necessary, as we qualitatively analysed patient symptoms and characteristics. The method used to prepare missing summary statistics of age range was to report the mean age.

### Evaluation methods

#### Computed tomography

Chest CT measures x-ray attenuation coefficients to determine lung tissue density and is routinely used in clinic to evaluate a variety of respiratory diseases, including but not limited to pneumonia, emphysema and interstitial lung disease. CT may be used to discern between pulmonary diseases and evaluate severity, as well as monitor disease progression and treatment response.

#### Positron emission tomography

PET is a quantitative molecular imaging modality that utilizes radiotracers to measure pulmonary ventilation, perfusion and blood flow as well as metabolic activity. The radiotracers used in PET imaging emit positrons, which will typically travel a few millimeters before colliding with an electron in the surrounding tissue, resulting in the emission of two 511 keV photons in opposite directions that are then detected by PET detectors oriented around the patient. In the context of lung disease, PET imaging is mostly commonly used to detect cancerous cells. However, PET radiotracers may also be used to evaluate the deposition of inhaled substances using carbon-11 (^11^C) and fluorine-18 (^18^F) (62), inflammation using ^18^F-fluorodeoxyglucose (^18^F-FDG) (63) and ventilation using nitrogen-13 (^13^N_2_) (63).

#### Pulmonary functional MRI

Hyperpolarized noble gas, oxygen-enhanced ^1^H, free-breathing ^1^H and arterial spin labelling methods are employed in order to acquire pulmonary functional MR images. These MRI techniques, in combination, may be used to measure lung ventilation and/or perfusion (64, 65), and are sensitive to early disease changes not easily detected with conventional pulmonary function tests (64, 66).

Hyperpolarized noble gas MRI allows for the visualization of the gas distribution *in vivo*, and is sensitive to the transitional and respiratory zones of the lung (67). Hyperpolarized ^3^He or ^129^Xe act as inhaled contrast agents, providing three-dimensional images with increased spatial and temporal resolution as compared to other functional imaging methods such as PET (68, 69), ^133^Xe scintigraphy (70), Xe-enhanced CT (71) and single photon emission computed tomography (SPECT) (68, 69). Ventilation abnormalities depicted on hyperpolarized MR images are associated with pulmonary structural abnormalities including luminal plugging, air trapping, airway inflammation and emphysema (68, 72–76). Hyperpolarized gas MRI has mainly been used as a research tool to investigate respiratory diseases including COPD (77), asthma (67), cystic fibrosis (78) and idiopathic pulmonary fibrosis (79). Recently, hyperpolarized ^129^Xe was approved by the US FDA for clinical use (80).

Oxygen-enhanced ^1^H MRI is a research technique that relies on the weak paramagnetic properties in oxygen, which decreases the longitudinal relaxation time (T1) of tissues, thus increasing the signal (81). By using O_2_ as a contrast agent, the presence and/or absence of ventilation may be evaluated and specific ventilation may be quantified (81). Oxygen-enhanced MRI has previously been used to investigate patients with COPD (82), interstitial lung disease (83) and asthma (84).

Free-breathing ^1^H MRI is also mainly a research tool which can be used to evaluate pulmonary ventilation and perfusion without the need for an endogenous contrast agent. This technique has been applied to patients with various cardiopulmonary diseases including COPD, CTEPH and asthma (85–88). These images are post-processed using non-rigid registration which compensates for respiratory motion, eliminating the need for patient compliance during a breathhold. Commonly used image processing techniques for free-breathing MRI include phase-resolved functional lung (PREFUL) (88) and matrix pencil decomposition (89).

Arterial spin labelling MRI can be used to evaluate pulmonary blood flow and its heterogeneity. This technique involves the acquisition of two images, which are subsequently subtracted to remove the blood signal from the tissue (90). This results in a perfusion-weighted image of the blood delivered within one cardiac cycle.

## Results

### Participants and report characteristics

**Figure 5:**
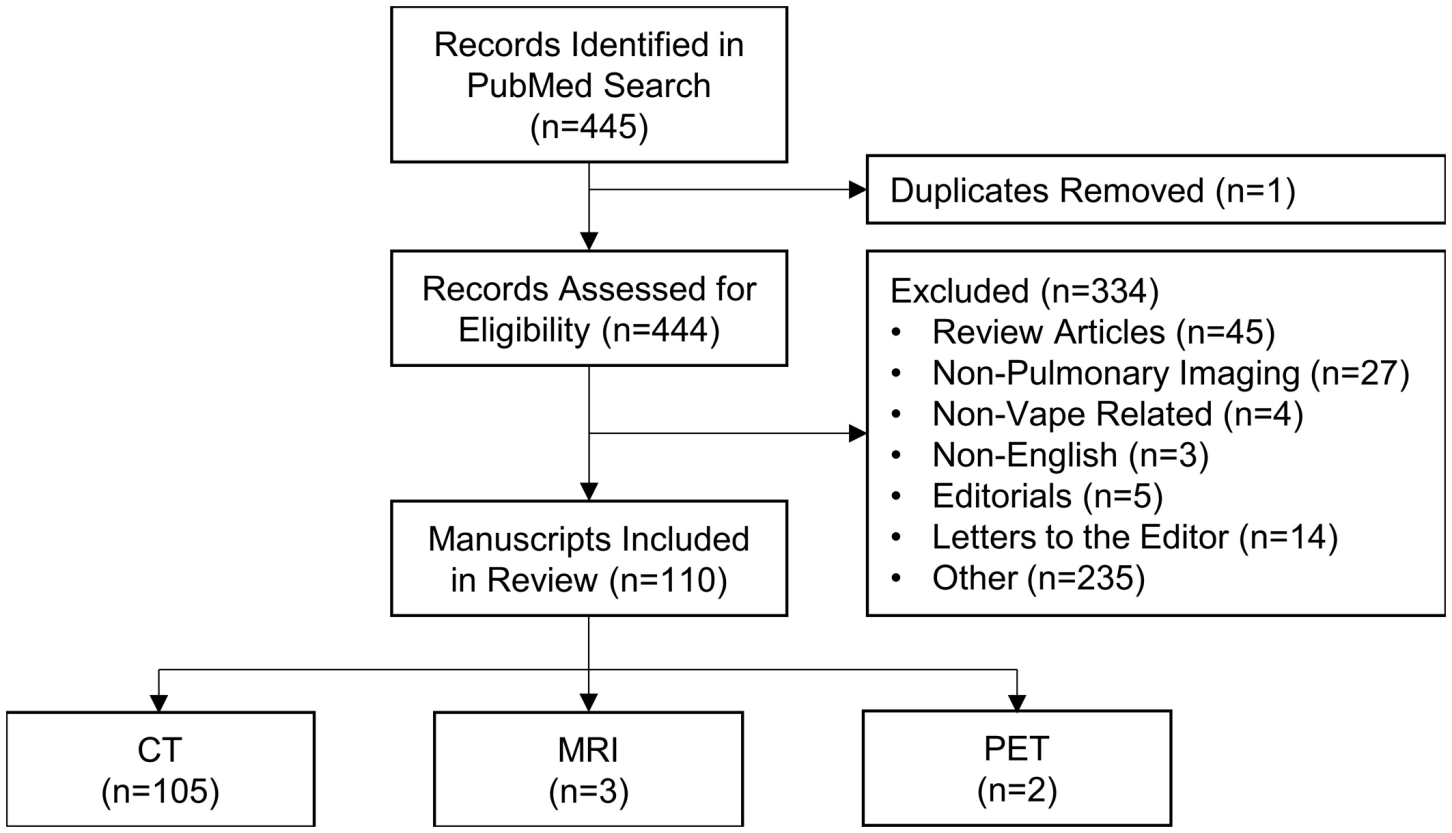
Flow chart of study search and selection process.

A flowchart is provided in Figure 5; Supplementary Figure S2 which show that the PubMed search conducted on June 132,023 yielded 445 manuscripts, one of which was identified as a duplicate and was excluded. The abstracts of the remaining 444 manuscripts were screened and an additional 235 manuscripts were excluded. Following a thorough full text evaluation, 45 review articles, 27 non-pulmonary articles, 15 letters to the editor, five editorials, four non-vape related and three non-English articles were excluded. Ultimately, 110 manuscripts met the inclusion criteria and were included in this review.

The characteristics of the included studies are listed in Supplementary Tables S1, S2. The vast majority of studies were conducted in North America (*n* = 90) with the remaining studies having been conducted in Europe (*n* = 15), Asia (*n* = 4) and South America (*n* = 1). A total of 668 e-cigarette users or patients that were exposed to vaping performed MRI, CT and/or PET scans; the primary imaging modality for 625 patients was CT, for 20 patients was PET and for 22 patients was MRI; a single participant was presented in two separate manuscripts, where one report described the clinical case and CT findings (91) and the other described research MRI and quantitative CT findings (61). In addition, each PET scan was accompanied by an anatomical CT. The minimum number of patients included in each study was 1 and the maximum was 160. Nearly all (*n* = 109) studies evaluated current or former e-cigarette users; one study (92) prospectively evaluated the acute effects of nicotine-based vaping in 15 healthy adults. The reported active ingredients in the e-liquid used by patients is summarized in Table 3. A total of 354 participants exclusively used nicotine (*n* = 101), THC (*n* = 247) or other substances (*n* = 6) in vape devices, 233 participants used both nicotine and either THC (*n* = 230) or containing other substances (*n* = 3) and 10 participants used a combination of three substances, while the contents of the vape devices of the remaining 71 participants was unspecified or unknown.

**Table 3:** Active ingredients in e-liquid reported in included studies.

Vape component *n* (%)	Manuscripts *n* = 110	Participants *n* = 668^a^
Nicotine	35 (32)	101 (15)
THC	46 (42)	247 (37)
**Dual**		
Nicotine + THC	39 (35)	230 (34)
Nicotine + other	3 (3)	3 (0.4)
**Triple**		
Nicotine + THC + other	3 (3)	10 (1)
Unspecified	29 (26)	71 (11)
Other	4 (4)	6 (1)

THC, Δ^9^-tetrahydrocannabinol.

a A single participant was described in two separate case studies.

### CT findings

Of the included manuscripts, CT was the most common imaging modality used to evaluate pulmonary abnormalities in vaping-related lung injury (105/110 manuscripts; 95%). Table 4; Supplementary Table S1 provide summaries of the findings from the included CT manuscripts. The majority of manuscripts reported on patients that were adolescents or young adults (61/105; 58%), with patient age ranging from 13 to 68 years old. Of the CT manuscripts included, 92 (88%) were case studies and 13 (12%) were retrospective studies. Acute lung injury was reported in 97 (92%) studies and 600 (98%) patients, chronic lung injury was reported in 7 (7%) studies and 13 (2%) patients, and both were reported in 2 (2%) studies. Nineteen (18%) studies involved participants who had previous respiratory diseases including: asthma (*n* = 15), tuberculosis (*n* = 1), allergic rhinitis (*n* = 1) and COPD (*n* = 1).

**Table 4:** CT findings.

CT study type & findings	Number of studies *N* = 105	Number of participants *N* = 612
**Study types**		
Cases	92 (88)	201 (33)
Retrospective	13 (12)	411 (67)
**Lung injury type**		
Acute lung injury	97 (92)	600 (98)
Chronic lung injury	7 (7)	12 (2)
Acute and chronic lung injury	1 (1)	24
**Major CT findings**		
Ground-glass opacities	84 (80)	347 (57)
Consolidation opacities	37 (35)	176 (29)
Subpleural sparing	32 (30)	230 (38)
Septal thickening	24 (23)	145 (24)
Patchy opacities	22 (21)	44 (8)
Pleural effusion	22 (21)	88 (14)
Pneumomediastinum	14 (13)	28 (5)
Lymphadenopathy	12 (11)	135 (22)
Bronchial wall thickening	10 (10)	45 (7)
Pneumothorax	10 (10)	12 (2)
Centrilobular nodules	9 (9)	72 (12)
Organizing pneumonia	9 (9)	42 (7)
Diffuse pulmonary nodules	7 (7)	8 (1)
Crazy paving	7 (7)	43 (7)
Pulmonary infiltrates	7 (7)	10 (2)
Tree in bud	5 (5)	7 (1)
Reverse halo sign	5 (5)	16 (3)
Bronchiolitis	5 (5)	9 (2)
Unspecified opacities	4 (4)	94 (15)
Emphysema	4 (4)	18 (3)
Parenchymal sparing	3 (3)	4 (1)
Peripheral sparing	3 (3)	3 (0.5)
Bullae	3 (3)	3 (0.5)
Pericardial effusions	3 (3)	5 (1)
Pneumonitis	3 (3)	7 (1)
Peribronchovascular sparing	3 (3)	3 (0.5)
Interlobular thickening	3 (3)	7 (1)
**Uncommon CT findings**		
Parenchymal opacities	2 (2)	25 (4)
Interstitial opacities	2 (2)	7 (1)
Honey combing	2 (2)	3 (0.5)
Prominent mosaicism	2 (2)	5 (1)
Bronchiectasis	2 (2)	4 (1)
Solid nodules	2 (2)	3 (0.5)
Bilateral patchy Infiltrates	2 (2)	92 (15)
Subpleural cysts	2 (2)	2 (0.3)
Interstitial thickening	2 (2)	2 (0.3)
Mosaic attenuations	2 (2)	5 (1)
Bronchocentric opacities	1 (1)	1 (0.2)
Bronchiolar dilation	1 (1)	9 (2)
Fissural displacement	1 (1)	6 (1)
Pulmonary embolism	1 (1)	1 (0.2)
Alveolar infiltrates	1 (1)	1 (0.2)
Necrotizing pneumonia	1 (1)	1 (0.2)
Tracheomalacia	1 (1)	1 (0.2)
Centrolobular thickening	1 (1)	1 (0.2)
Peripheral opacities	1 (1)	5 (1)
Peribronchovascular opacities	1 (1)	6 (1)
Miliary pattern	1 (1)	1 (0.2)

Ground-glass opacities, defined as a hazy increase in lung density observed on chest CT (93), were the most common CT finding in the included manuscripts, with 84 (80%) studies and 347 (57%) participants reporting evidence of this pulmonary abnormality. Consolidation opacities (37 studies/176 participants), subpleural sparing (32 studies/230 participants), septal thickening (24 studies/145 participants) and patchy opacities (22 studies/44 participants) were also common.

**Figure 6:**
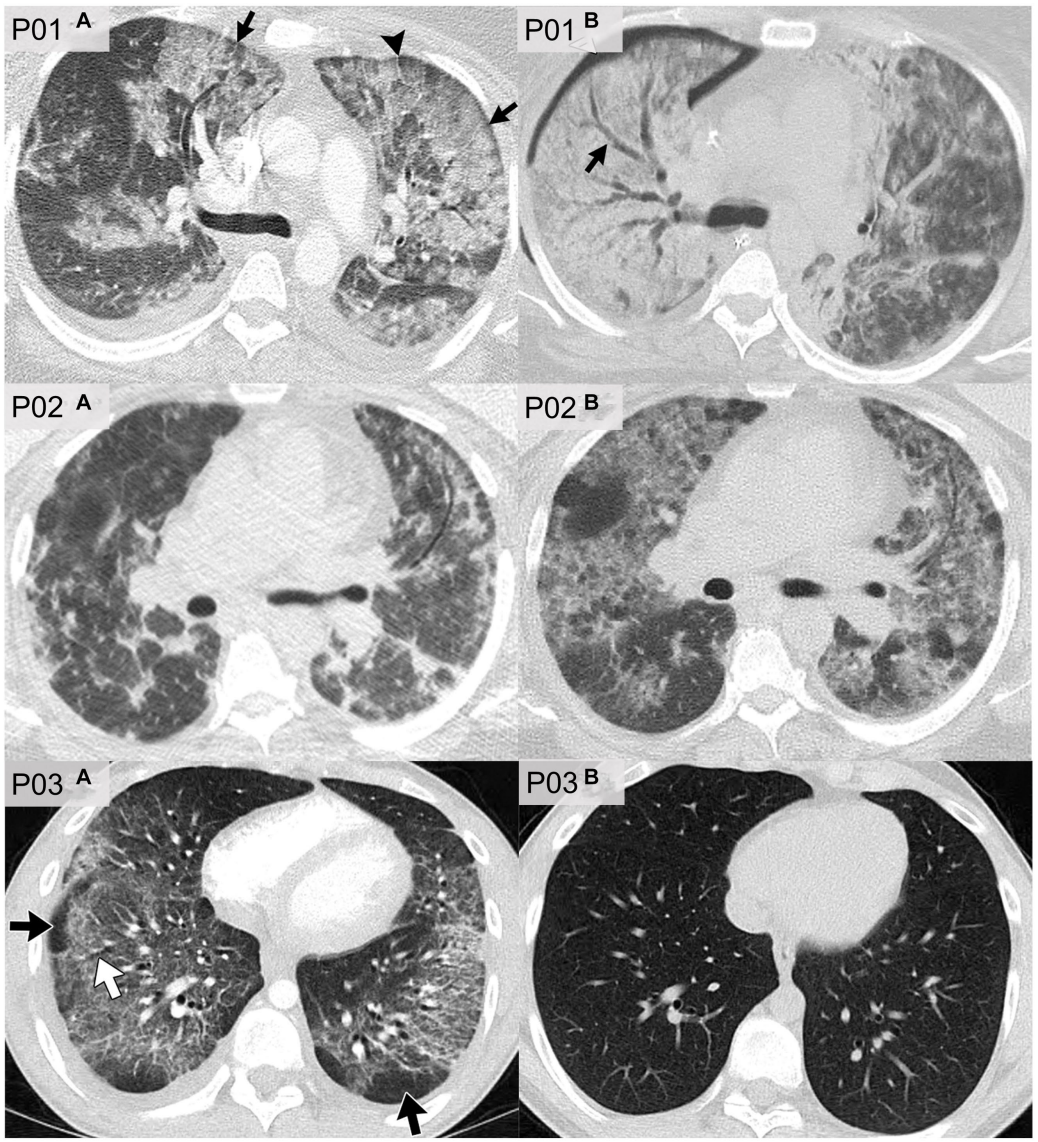
CT imaging of e-cigarette-related or vaping-associated lung injury (EVALI) with short-term follow-up. P01: axial CT of a 35 years-old female with diffuse alveolar damage pattern who vaped THC. **(A)** CT imaging showed ground-glass opacities with areas of consolidation, subpleural and perilobular sparing (arrows) and septal thickening (arrowhead). **(B)** CT 2 weeks later showed extensive right lung consolidation with areas of bronchial dilation (arrow) and internal development of right pneumothorax. Patient died 5 days later. P02: axial CT of a 51 years-old female showing multiple episodes of EVALI following repeated vaping of nicotine with mint flavoring. **(A)** CT imaging showed scattered areas of ground-glass opacities with subpleural sparing. **(B)** Two months later, the patient returned to emergency department with dyspnea and fever. CT findings included more extensive ground-glass opacities with areas of lobular and subpleural sparing. Septal thickening is present creating “crazy paving” pattern. Patient’s condition deteriorated, and was complicated by aspiration pneumonia and bilateral lower-lobe collapse. P03: axial CT of organizing pneumonia pattern in a 20 years-old male who vapes nicotine and THC products daily. **(A)** CT imaging showed peribronchiolar ground-glass opacities with subpleural sparing (black arrows). Areas of bronchial dilation are seen in areas of ground-glass opacities (white arrow). **(B)** Four weeks following steroid therapy, the patient’s CT scan was normal. Images reproduced with permission from Kligerman et al. (40).

Figure 6 shows axial CT slices of three patients, each at two separate time-points, previously published and described by Kligerman et al. (94). P01 was a 35 years-old female who reported the use of THC-based e-cigarettes and who presented with CT evidence of ground-glass opacities and consolidations as well as subpleural and perilobular sparing (P01a). CT images that were acquired 2 weeks later (P01b) showed extensive consolidation along with areas of bronchial dilation and the development of a right pneumothorax; this patient died 5 days later. P02 was a 51 years-old female who reported the use of nicotine-based e-cigarettes and who presented with CT evidence of ground-glass opacities and subpleural sparing (P02a). Two months later (P02b), CT images revealed more extensive ground-glass opacities and septal thickening which presented as “crazy paving” pattern. P03 was a 20 years-old male who reported the use of both THC- and nicotine-based e-cigarettes. CT imaging (P03a) in this patient revealed organizing pneumonia, peribronchiolar ground-glass opacities and subpleural sparing. CT imaging 4 weeks later (P03b) normalized, after the patient was treated with steroid therapy.

Table 5 summarizes CT findings from the 13 included retrospective studies. All patients described in these studies presented with probable or confirmed EVALI. Six of these studies reported on pediatric patients and four reported findings in adult patients, while the remaining three described findings in both pediatric and adult populations.

**Table 5:** Retrospective CT manuscripts summary.

Author	Objective	Location	Summary
Aberegg et al. (95)	Describe findings and outcomes of EVALI	Salt Lake City, United States	Patients commonly presented with organizing pneumonia pattern on CT Radiographic opacities resolved within 30 days
Artunduaga et al. (96)	Evaluate chest radiographic and CT findings of EVALI	Dallas, United States	EVALI characterized by bilateral ground-glass opacities, consolidation on CT
Carroll et al. (97)	Evaluate short-term outcomes in EVALI	Milwaukee, United States	Ground-glass and patchy opacities common
Chidambaram et al. (98)	Present clinical and imaging findings in adolescents with respiratory symptoms	Philadelphia, United States	Imaging findings include ground-glass opacities, subpleural sparing and basilar opacities
Harry-Hernandez et al. (99)	Define pathologic findings in patients with EVALI	Multicenter—United States	Pathologic BAL and biopsy findings correlated with CT findings
Helfgott et al. (100)	Report on radiological findings in adolescents with EVALI and COVID-19 symptoms	New Brunswick, United States	CT findings of EVALI similar to COVID-19
Kalininskiy et al. (101)	Summarize clinical presentation of patients with probable or confirmed EVALI	Rochester, United States	CT findings were resolved at follow-up
Kligerman et al. (94)	Describe frequency of imaging findings in EVALI	Multicenter—United States	Ground-glass opacities, and subpleural, lobular and peribronchovascular sparing were common Increased vaping frequency associated with more severe injury
Layden et al. (102)	Summarize clinical characteristics of EVALI patients	Wisconsin and Illinois, United States	Bilateral infiltrates on imaging in all patients Ground-glass opacities and subpleural sparing were common
Pajak et al. (103)	Describe imaging findings in EVALI	Delaware, United States	Subpleural sparing, opacities and consolidation were common
Panse et al. (104)	Describe CT patterns in EVALI	Arizona, United States	EVALI acutely presents as ground-glass opacity and consolidation Longitudinal CT pattern resembles subacute hypersensitivity pneumonitis
Rao et al. (105)	Describe diagnosis, evaluation and management of EVALI in adolescents	Dallas, United States	All patients demonstrated bilateral ground-glass opacities on CT
Wang et al. (106)	Describe most common CT findings in EVALI	Houston, United States	Opacities with subpleural and peribronchovascular sparing commonly observed

EVALI, e-cigarette-related or vaping-associated lung injury; CT, computed tomography; BAL, bronchoalveolar lavage.

Nearly all (10/13) of the retrospective studies reported that ground-glass opacities, subpleural sparing and/or consolidation were among the most common CT findings in patients with EVALI (94–98, 102–106). Three investigations included short-term (within 30 days of initial presentation) follow-up imaging (95, 101, 104). Kalininskiy et al. (101) reported that CT abnormalities in patients treated with antibiotics were largely resolved within 17 days of their initial emergency department visit; Aberegg et al. (95) also reported resolution of CT opacities after treatment with antibiotics and/or corticosteroids. CT abnormalities cleared in some patients described by Panse et al. (104) despite extensive injury at initial presentation, although some patients described showed evidence of residual imaging findings. Prospective longitudinal imaging studies will help elucidate whether such residual abnormalities are the result of slow healing areas of injury or point to the development of permanent scarring.

### PET findings

Table 6 summarizes the included PET studies (*n* = 2). In total, 20 patients were either current or former e-cigarette users, or were acutely exposed to e-cigarettes.

**Table 6:** Manuscripts with PET and MRI endpoints.

Author	Objective	Location	Summary
Eddy et al. (61)	Evaluate teenage male post ECMO for EVALI	London, Canada	Persistent, chronic, irreversible airflow limitations and gas trapping requiring mechanical ventilation and ECMO Continuous abnormal ventilation pattern dissimilar to ventilation heterogeneity observed in asthma or COPD
Kizhakke Puliyakote et al. (107)	Assess ventilation-perfusion mismatch in asymptomatic e-cigarette users using MRI to determine a correlation	San Diego, United States	Impaired ventilation-perfusion matching in the lung through an alteration of both ventilation and perfusion Degree of disruption after a session matches that of patients with COPD
Nyilas et al. (108)	To examine the immediate effect of ENDs exposure and tobacco smoke on lung ventilation and perfusion by functional MRI and lung function tests	Bern, Switzerland	Local perfusion increased in participants who used ENDS after exposure No change in perfusion was detected in the group of participants who used nicotine-free e-liquids No change in lung function compared to baseline was observed (nicotine and non-nicotine) Ventilation perfusion mismatch in ENDS users
Wall et al. (92)	Investigate the distribution and deposition of inhaled [^11^C] nicotine using freebase nicotine and lactate salt nicotine	Uppsala, Sweden	Freebase nicotine exhibited higher uptake and deposition in the upper respiratory pathways Lactate nicotine showed lower tracer uptake and accumulation in the upper respiratory pathways and an earlier peak and a steeper decline in the lung
Wetherill et al. (109)	Used PET to quantify inducible nitric oxide synthase expression to characterize oxidative stress and inflammation in the lungs *in vivo*	Pennsylvania, United States	ENDS users showed greater ^18^F-NOS non-displaceable binding potential (BP_ND_) than cigarette smokers (*p* = 0.03) and controls (*p* = 0.01) ^18^F-NOS lung tissue delivery and inducible nitric oxide synthase distribution volume did not significantly differ among groups

ECMO, extracorporeal membrane oxygenation; EVALI, e-cigarette-related or vaping-associated lung injury; COPD, chronic obstructive pulmonary disease; MRI, magnetic resonance imaging; ENDS, electronic nicotine delivery systems; PET, positron emission tomography.

**Figure 7:**
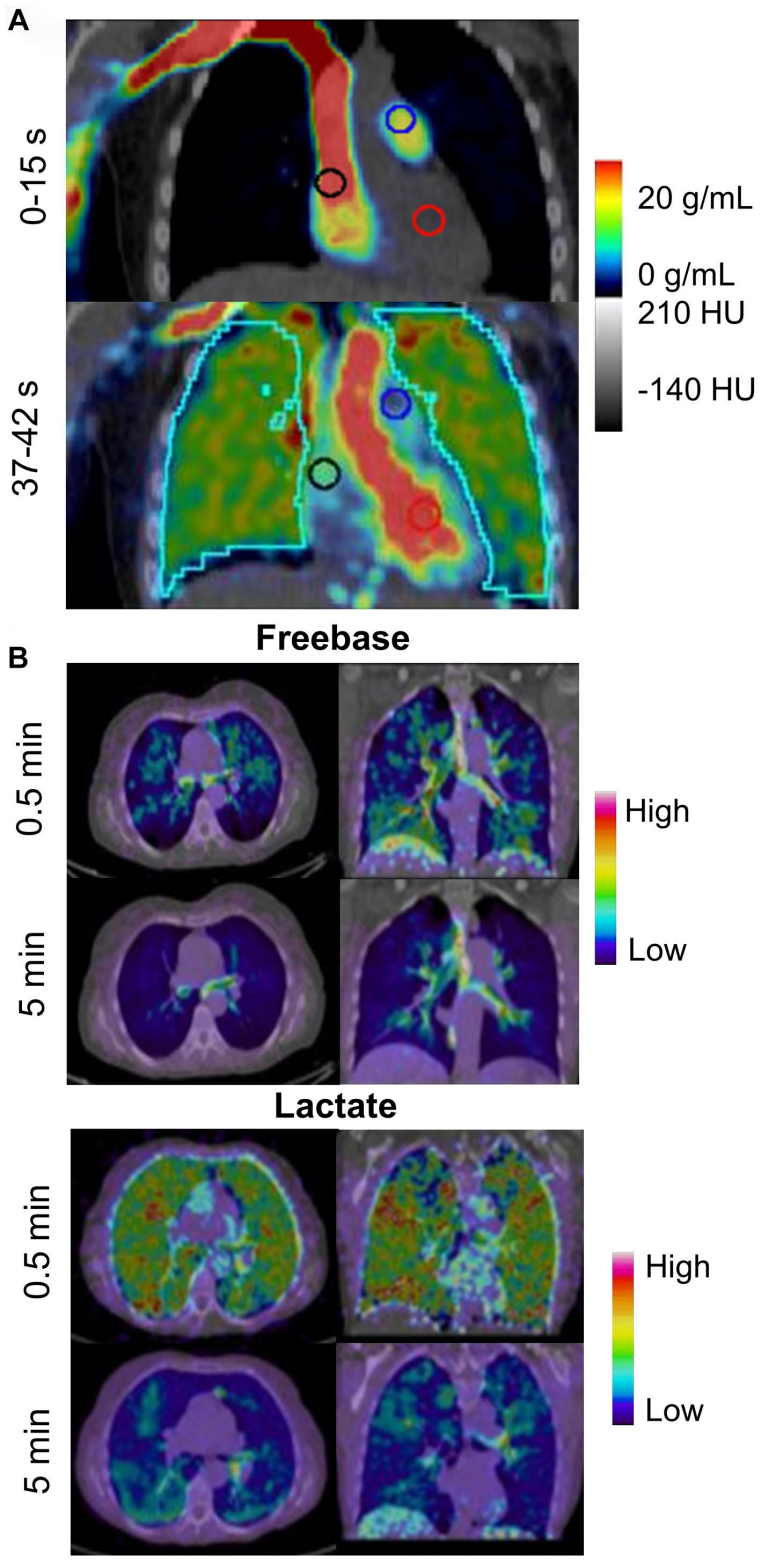
PET imaging tracking metabolic activity in e-cigarette users and smokers. **(A)** Oronal PET/CT ^18^F-NOS in an e-cigarette user (unspecified active ingredient). Uptake is shown 0–15 s and 37–42 s after injection of ^18^F-NOS to quantify oxidative stress and inflammation in the lungs. Adapted from Wetherill et al. (109) under Creative Commons License. **(B)** Representative distribution of ^11^C-nicotine freebase and lactate in lungs at 0.5 and 5 min after inhalation of the tagged nicotine salts through an e-cigarette in a healthy adult smoker. Adapted from Wall et al. (92) under Creative Commons License.

Wetherill et al. (109) used the radiotracer ^18^F-nitric oxide synthase (NOS) to characterize oxidative stress and inflammation in the lungs of e-cigarette users (*n* = 5) and compare with combustible cigarette smokers (*n* = 5) and healthy controls (*n* = 5). They revealed that e-cigarette users showed greater non-displaceable binding potential (Figure 7A) than combustible cigarette smokers and healthy controls, which is associated with the pro-inflammatory cytokine TNF-alpha that is involved in the inflammatory cascade of acute lung injury. They also observed that pulmonary inflammation was greater in e-cigarette users than combustible cigarette smokers and healthy controls.

In contrast, Wall et al. (92) investigated the acute deposition of inhaled ^11^C-nicotine in 15 healthy adults using two nicotine formulations—freebase and lactate salt. In this study, the authors showed that freebase nicotine exhibited high uptake and deposition in the upper respiratory pathways while lactate nicotine was deposited throughout the entire lung and bronchial tree (Figure 7B). Lactate nicotine was also distributed more rapidly than freebase nicotine.

### MRI findings

Table 6 also summarizes the included MRI reports (*n* = 3). A total of 23 patients who were current or former e-cigarette users were reported.

**Figure 8:**
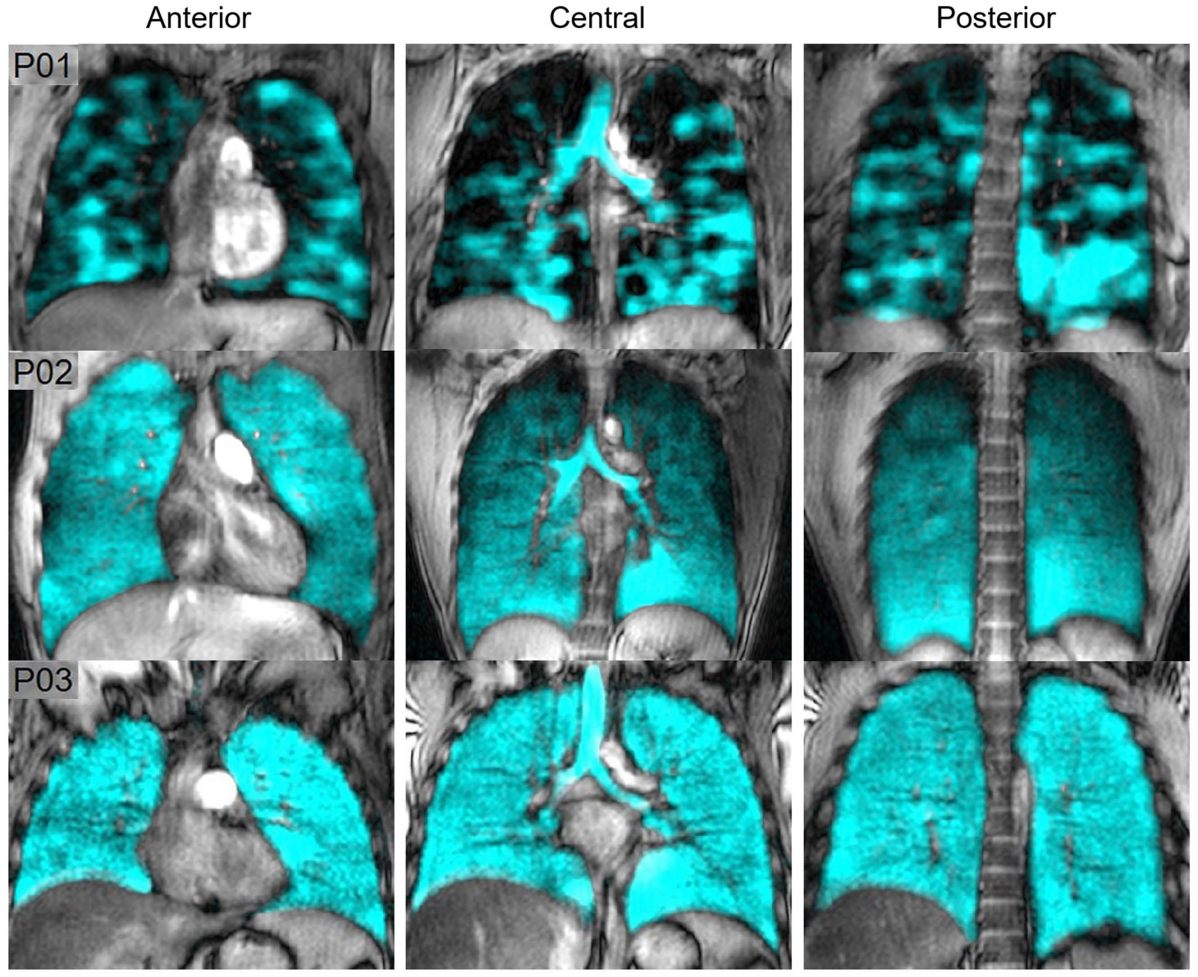
Hyperpolarized ^129^Xe MR ventilation imaging in chronic vapers and similar-aged healthy volunteer. Anterior, central, and posterior coronal slices of ^129^Xe ventilation (cyan) co-registered with anatomical ^1^H (greyscale) MRI. All images were acquired by the authors’ group. P01 is an 18 years-old male with severe bronchiolitis and respiratory failure caused by e-cigarette use, 1 month post discharge after a 6 months history of vaping (VDP = 21%). P02 is a 29 years-old male with a 2.5 years history of vaping nicotine (3/4 pods daily) (VDP = 4.7%). P03 is a 22 years-old male with no history of chronic respiratory abnormalities, vaping or combustible cigarette use (VDP = 1.5%).

Figure 8 shows hyperpolarized ^129^Xe MRI ventilation (cyan) co-registered with anatomical ^1^H MRI (greyscale) for one participant with EVALI, one asymptomatic e-cigarette user and one healthy participant; all images were acquired by the authors’ group. The participant with EVALI demonstrates a heterogenous ventilation pattern with multiple ventilation defects, represented as black regions, throughout the entire lung. In contrast, the asymptomatic e-cigarette user demonstrates a relatively homogenous ventilation pattern with evidence of small, peripheral ventilation defects in the lung apices. Finally, the healthy participant has a homogenous ventilation distribution with no ventilation defects.

Two studies compared the acute effects of e-cigarette use on ventilation. In the first, alveolar ventilation and ventilation heterogeneity in asymptomatic e-cigarette users were similar to healthy controls prior to exposure, however both measurements worsened following exposure to vaping in e-cigarette users (107). In contrast, while the second study observed a nominal increase in ventilation impairment post-exposure, this change was not statistically significant (108). In a similar manner, the ^129^Xe MRI ventilation pattern of a patient recovering from EVALI was highly abnormal and dissimilar to the patterns previously observed in either asthma or COPD (61). Furthermore, these ventilation abnormalities persisted for at least 8 months (61).

**Figure 9:**
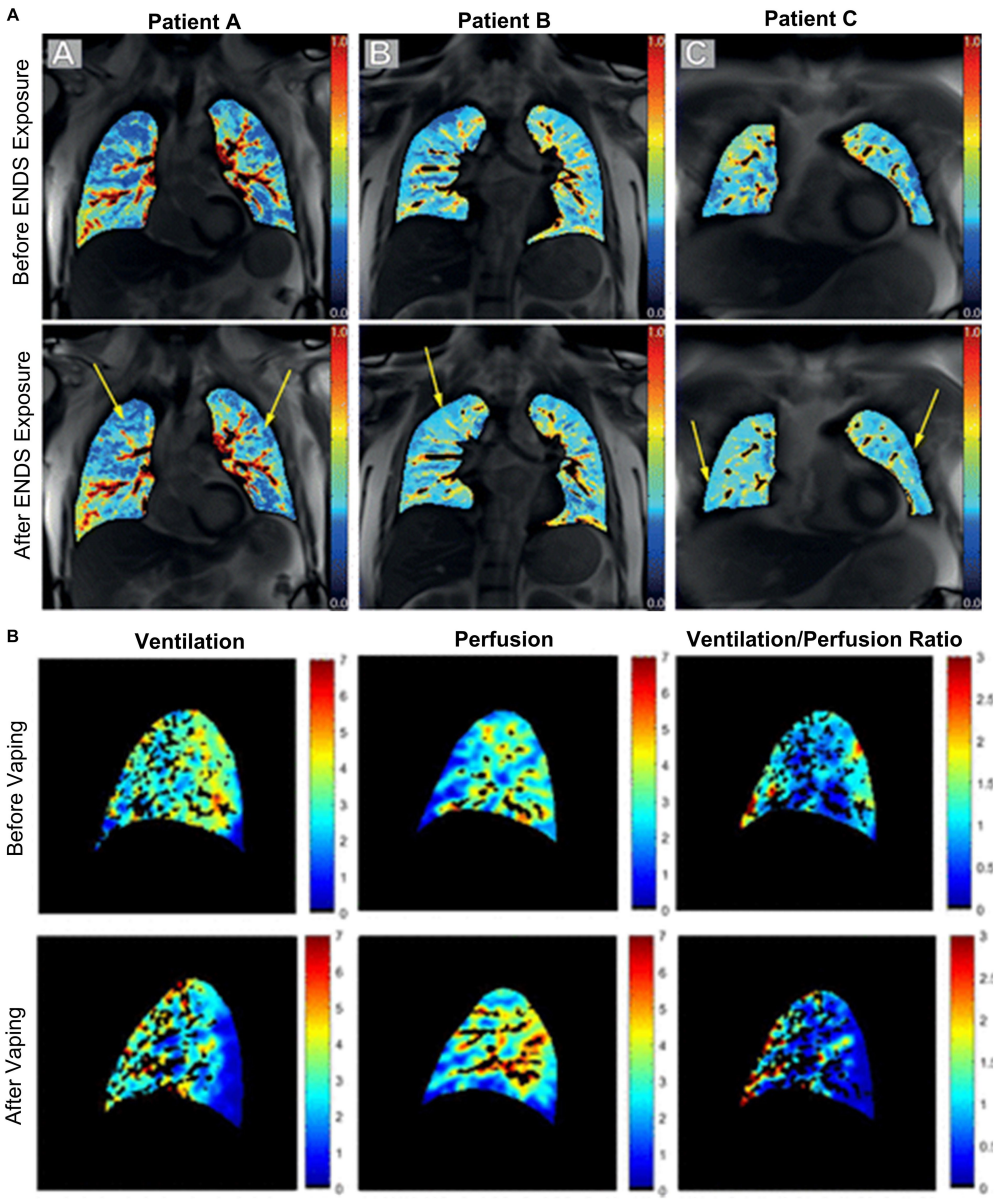
Free-breathing ^1^H MRI of acute effects of vaping. **(A)** Pulmonary perfusion images obtained by using non-contrast matrix pencil MRI in three electronic nicotine vape users before and after exposure. The arrows indicate lung regions with increased regional perfusion post-exposure. Red corresponds to greater perfusion amplitude and blue corresponds to lower values (108). **(B)** Spatial maps of ventilation, perfusion, and ventilation-perfusion ratios, before and after vaping, in a single sagittal slice of one representative subject. Black regions within the lung field represent regions excluded from analysis, including conducting airways and vessels, and regions with poor signal to noise (107).

MRI perfusion maps are shown for four e-cigarette users in Figure 9, demonstrating changes in regional perfusion following exposure to e-cigarettes. Two studies have evaluated pulmonary perfusion in e-cigarette users prior to and following acute exposure to e-cigarettes and both observed similar findings. Perfusion prior to exposure was similar between healthy controls and e-cigarette users, and following exposure, perfusion was increased (107) while perfusion heterogeneity was decreased (108). Furthermore, e-cigarette users who were exposed to nicotine-based e-liquids demonstrated significant increases in perfusion while those who were exposed to nicotine-free e-liquids were not. Finally, ventilation/perfusion heterogeneity was increased throughout the lungs of e-cigarette users as compared to healthy controls, both prior to and following acute exposure to e-cigarettes (107); the extent of this disruption in ventilation/perfusion matching was similar to what has previously been observed in COPD.

## Discussion

Despite nearly 3,000 EVALI cases having been reported in the US in less than 12 months in 2019, the impact of e-cigarette use or vaping on respiratory health is still largely unknown. This is, in part, due to a lack of prospective studies designed to sensitively evaluate the pathological mechanisms responsible for the development of this disease. In this systematic review we summarize in e-cigarette users and patients with EVALI the following points: (1) CT sensitively revealed pulmonary structural abnormalities, (2) PET measured pulmonary inflammation and the deposition of e-cigarette aerosolized particles, and (3) ventilation and perfusion measured via MRI was abnormal. These findings demonstrated that pulmonary functional and structural abnormalities were common in both patients with vaping-induced lung injury and in e-cigarette users with or without respiratory symptoms, and that these abnormalities may be sensitively measured using CT, PET and pulmonary functional MRI. Furthermore, these findings support the use of imaging modalities in prospective studies to help uncover the pathological drivers and mechanisms underlying respiratory symptoms and the development of EVALI in previously healthy adolescents and young adults.

Our systematic review included any report which used CT, PET or MRI to evaluate patients with EVALI or e-cigarette users. CT was the most commonly reported imaging modality, with 105 of the 110 included studies primarily reporting CT findings. Ground-glass opacities, consolidation opacities, subpleural sparing, septal thickening and patchy opacities were among the most common CT findings, in patients with EVALI and e-cigarette users. These findings demonstrated that CT is highly useful in the diagnosis and monitoring of patients with EVALI. Moreover, the worsening or resolution of CT abnormalities, with or without therapeutic intervention, may help inform on the pathological drivers of EVALI, point towards potential treatment options or help identify characteristics of patients at risk of developing serious and/or permanent lung injury as a result of e-cigarette use.

PET showed that there were differences in the deposition distribution of freebase or lactate salt nicotine aerosolized particles (92). This is important because early e-liquids primarily used freebase nicotine however, since 2017, lactate salt nicotine is more commonly used (110). The health risks of lactate salt and freebase nicotine have not yet been studied. PET also revealed a unique inflammatory response to acute e-cigarette exposure, in comparison with acute combustible cigarette exposure or in healthy controls. These findings point towards possible mechanisms of acute lung injury following e-cigarette use. Together, these preliminary studies may drive further hypothesis-driven research into the pulmonary health effects associated with e-cigarette use.

Unlike CT and PET, pulmonary functional MRI does not require ionizing radiation and multiple acquisitions can be completed over either short or long periods of time. In the context of vaping, pulmonary functional MRI sensitively revealed ventilation and perfusion abnormalities in e-cigarette users following acute exposure to e-cigarettes (107, 108), and in a patient recovering from EVALI (61). Ventilation abnormalities in e-cigarette users may be driven by airflow obstruction via inflammation, impaired mucus clearance, constriction or collapse (107). In addition, perfusion abnormalities were worse in patients using nicotine-based e-liquids as compared to those using nicotine-free e-liquids, which points towards known impacts of nicotine on cardiovascular hemodynamics, increases in heart rate, systolic blood pressure and cardiac output (107). Furthermore, findings of increased perfusion and decreased perfusion heterogeneity in e-cigarette users, regardless of e-liquid content, may suggest hypoxic vasoconstriction as a compensatory mechanism in the presence of abnormal ventilation (107). This idea is further supported by the observation of increased ventilation/perfusion heterogeneity. Together, these findings show that MRI may be used to sensitively measure both the acute and chronic effects of e-cigarette use on both pulmonary ventilation and perfusion.

### Limitations

The reviewed manuscripts have a number of important limitations. First, our search criteria were quite specific and thus we may have excluded relevant studies which did not use our precise terminology. Second, technical CT parameters were not commonly described. The variation of CT parameters between studies and patients may have impacted the radiologist’s findings and thus, it may be possible that some CT abnormalities were undetected. Third, the commercialization and distribution of e-cigarette devices is recent and thus, the majority of the included studies reported on data acquired within the past 4 years. It is not possible at this time to comment on the long-term health effects associated with either continuous or sporadic e-cigarette use as this has not yet been investigated and/or reported. Fourth, a large proportion (26% of studies and 11% of patients) of the included studies did not report or were not able to ascertain the specific ingredients present in the e-liquids used by the studied patients. With the wide variety of e-liquids available, as well as the potential combination of multiple e-liquids by users, the effect of individual or combinations of e-liquids and their ingredients on respiratory health is largely unknown. Finally, e-cigarette devices have evolved and many components of these may be customized, including the voltage of the battery, the e-liquid temperature, nicotine yield or puff volume (111–113), introducing a wide number of variables that may contribute to vaping-induced lung injury.

## Conclusion

With the growing popularity of e-cigarettes for recreational use in people who have not previously smoked combustible cigarettes, it is important to understand the short- and long-term effects of vaping on respiratory health. This systematic review, while revealing only a handful of functional imaging studies, showed that pulmonary imaging and in particular functional imaging is helpful in generating a better understanding of the acute and chronic effects of vaping on pulmonary structure and function. The time is right to explore larger scale, multi-centre studies using pulmonary functional imaging methods to uncover the pathological mechanisms driving vaping-induced lung injury and respiratory symptoms, and to evaluate interventions.

## Data availability statement

The original contributions presented in the study are included in the article/Supplementary material, further inquiries can be directed to the corresponding author.

## Author contributions

JH: Data curation, Formal analysis, Investigation, Visualization, Writing – original draft. VP: Data curation, Formal analysis, Investigation, Visualization, Writing – original draft. JZ: Data curation, Formal analysis, Investigation, Visualization, Writing – original draft. MS: Supervision, Visualization, Writing – review & editing. GP: Conceptualization, Investigation, Methodology, Supervision, Writing – review & editing. MM: Supervision, Visualization, Writing – review & editing.
