# Supplementary material for: Review of quantitative and functional lung imaging evidence of vaping-related lung injury
Source: Front Med (Lausanne). 2024 Jan 24;11:1285361. doi: 10.3389/fmed.2024.1285361 (PMC10847544; doi:10.3389/fmed.2024.1285361)
Supplement: Supplementary file 1 [file Data_Sheet_1.pdf]

REVIEW OF QUANTITATIVE AND FUNCTIONAL LUNG IMAGING EVIDENCE OF VAPING-RELATED LUNG INJURY

Supplementary Materials

Table S1. CT report characteristics

Manuscript	Study Type	Number of Participants	Age of Participants	Location	Symptoms
Abbara et al., 2019	Case study	n=1	24	USA	Chest pain, cough, fever
Aberegg et al., 2020	Retrospective	n=31	21-31	USA	Chills, cough, dyspnea, fever, nausea, vomiting
Adhikari et al., 2021	Case study	n=1	20	USA	Chest pain
Ahmad M., et al., 2020	Case series	n=2	23-46	USA	Cough, dyspnea, fever
Aldy et al., 2020	Case study	n=1	16	USA	Abdominal pain, chest pain, chills, fever, nausea, vomiting
Amin et al., 2020	Case study	n=1	17	USA	Abdominal pain, cough, dehydration, fever, intermittent diarrhea, vomiting
Anderson et al., 2021	Case study	n=1	16	USA	Cough, dyspnea, fever
Ansari-Gilani et al., 2020	Case series	n=3	18-64	USA	Cough, dyspnea, fever, intermittent diarrhea, nausea
Antwi-Amoabeng et al., 2020	Case study	n=1	20	USA	Cough, dyspnea, wheezing
Artunduaga et al., 2020	Retrospective	n=14	13-17	USA	EVALI symptoms
Billa et al., 2019	Case series	n=3	15-17	USA	Cough, dyspnea, hypoxemia, fever,
Borchert et al., 2021	Case report	n=1	34	Germany	Back pain, chest pain, dyspnea, dizziness, retrosternal pain
Bozkanat et al., 2020	Case study	n=1	17	USA	Abdominal pain, diarrhea, dyspnea
Carroll et al., 2019	Retrospective	n=15	17.1*	USA	Subacute pulmonary, gastrointestinal and constitutional complaints
Casamento Tumeo et al., 2022	Case study	n=1	15	Italy	Dyspnea
Cedeno et al., 2020	Case study	n=1	52	USA	Persistent dyspnea
Chapman et al., 2020	Case study	n=1	62	UK	Dyspnea, hemoptysis, reduced exercise tolerance
Chawla et al., 2020	Case study	n=1	15	USA	Cough, fatigue, vomiting
Chen et al., 2020	Case series	n=3	33-50	USA	Diarrhea, dyspnea, fever, headache, nausea, vomiting
Chidambaram et al., 2020	Retrospective	n=11	16.6*	USA	Progressive subacute respiratory distress with abdominal pain
Colesar et al., 2021	Case Study	n=1	18	USA	chest tightness, cough, trouble breathing

Collins et al., 2022	Case study	n=1	18	UK	Cough, progressive dyspnea
Conuel et al., 2019	Case series	n=5	23-55	USA	Dyspnea, hypoxia, fatigue
Corcoran et al., 2020	Case series	n=7	15-18	USA	Chest pain, cough, dyspnea, fever, nausea, vomiting
Cruz-Vidal et al., 2020	Case series	n=6	16-18	USA	Abdominal pain, chest pain, cough, diarrhea, dyspnea, nausea, fever, vomiting
Darmawan et al., 2020	Case series	n=3	16-17	USA	Chest pain, cough, dyspnea, fever, hypoxia, nausea, tachycardia, vomiting
Decarli et al., 2020	Case study	n=1	18	USA	Dyspnea, nausea, vomiting
Deliwala et al., 2020	Case study	n=1	41	USA	Cough, dyspnea
Deskins et al., 2022	Case study	n=1	15	USA	Chest pain, chest numbness
Dicpinigaitis et al., 2020	Case study	n=1	28	USA	Dry cough, diarrhea, dyspnea, intermittent sputum production, nausea
Drabkin et al., 2019	Case study	n=1	19	USA	Chest tightness, cough, fever
Edmonds et al., 2020	Case study	n=1	31	USA	Cough, hemoptysis
El Chebib et al., 2020	Case study	n=1	15	USA	Cough, diarrhea, dyspnea, fever, vomiting
Freathy et al., 2020	Case study	n=1	27	USA	Dyspnea
Fryman et al., 2020	Case series	n=8	24-62	USA	Cough, dyspnea, fever, headache
Ganne et al., 2021	Case report	n=1	32	USA	Abdominal pain, cough, dyspnea, fatigue, fevers, nausea, headaches
Girvin et al., 2020	Case series	n=6	19-46	USA	Chills chest pain, cough, fatigue, fever, diarrhea, dyspnea, nausea
Guarino et al., 2021	Case study	n=1	59	Italy	Progressive dyspnea on exertion
Hallowell et al., 2019	Case study	n=1	20	USA	Chills, dyspnea, fatigue, fever, sputum production
Harada et al., 2022	Case study	n=1	20	USA	Dyspnea, hemoptysis, fevers, nausea, severe vomiting
Haran et al., 2019	Case report	n=1	24	USA	Dyspnea, hemoptysis, nausea
Hariri et al., 2023	Case series	n=4	25-65	USA	Chest pain, dyspnea, fatigue
Harry-Hernandez et al., 2022	Retrospective	n=21	16-68	USA	EVALI symptoms

Helfgott et al., 2022	Retrospective	n=12	14-19	USA	Abdominal pain, chest pain, cough, diaphoresis, diarrhea, dyspnea, hypoxemia, fever nausea, vomiting
Hoshina, 2021	Case report	n=1	20	USA	Dyspnea, fever
Jankharia et al., 2020	Case study	n=1	43	India	Cough, cold, wheezing, disturbed sleep
Kalantary et al., 2021	Case study	n=1	Mid 60s	USA	Diarrhea, fevers, mild nausea, wheezing
Kalininskiy et al., 2019	Retrospective	n=12	21-35	USA	Cough, dyspnoea, fever, nausea
Kalra et al., 2020	Case study	n=1	34	USA	Chest pain, dyspnea, fevers
Kaous et al., 2020	Case series	n=8	21-34	USA	Chest pain, dyspnea, nausea, fever, vomiting
Kartiko et al., 2023	Case study	n=1	26	USA	Chest pain, throat pain
Kass et al., 2020	Case series	n=10	14-19	USA	Cough, diarrhea, dyspnea, hemoptysis, fever, nausea, vomiting, chest tightness, night sweats, weight loss
Kelley et al., 2020	Case report	n=1	17	USA	Abdominal pain, diarrhea
Kichloo et al., 2020	Case report	n=1	31	USA	Dry cough, dyspnea, fatigue, fever
Kligerman et al., 2021	Retrospective	n=160	15-68	USA	chest pain, cough, diarrhea, dyspnea, nausea
Kooragayalu et al., 2019	Case report	n=1	26	USA	Cough, dyspnea, fever
Kwack et al., 2023	Case study	n=1	34	South Korea	Dyspnea, mild fever
Landman et al., 2019	Case study	n=1	17	Canada	Acute bronchiolitis
Layden et al., 2020	Retrospective	n=98	21*	USA	Chest pain, cough, diarrhea, dyspnea, nausea, vomiting, fever
Lee et al., 2021	Retrospective	n=8	16-18	USA	Cough, nausea, fever, vomiting, weight loss, respiratory distress
Lim et al., 2021	Case study	n=1	24	South Korea	Acute dyspnea, fever
Lim et al., 2022	Case study	n=1	49	USA	Dyspnea
Lin et al., 2020	Case report	n=1	34		
Lu et al., 2020	Case study	n=1	17	USA	Chest pain, dyspnea, tachypnea
Lucas et al., 2023	Case Study	n=1	43	Brazil	Chills, coughing up sputum, fever, wheezing
Lucero et al., 2021	Case study	n=1	23	USA	Cough, fevers, vomiting
MacMurdo et al., 2020	Case series	n=15	19-60	USA	Abdominal pain, chest tightness, chills, cough, diarrhea, dyspnea, fever, night sweats

Maslonka et al., 2020	Case study	n=1	23	USA	Cough, dyspnea, fevers, intermittent confusion
Massey et al., 2021	Case study	n=1	33	USA	Dyspnea, fatigue, weight loss, night sweats
Messina et al., 2020	Case study	n=1	17	USA	Headache, fever, nausea
Messina et al., 2020	Case series	n=6	17-20	USA	Constitutional, gastrointestinal, neurologic and respiratory complaints
Mittal et al., 2020	Case study	n=1	53	USA	Dyspnea, wheezing
Mughal et al., 2020	Case study	n=1	57	USA	Abdominal pain, dry cough, dyspnea on exertion
Mukhopadhyay et al., 2020	Case series	n=8	19-61	USA	Cough, dizziness, dyspnea, fever, night sweats, weight loss, nausea, vomiting
Mull et al., 2020	Case study	n=1	16	USA	abdominal discomfort, chest pain, cough, diarrhea, dyspnea, fever
Mull et al., 2020	Retrospective	n=3	16-18	USA	Acute hypoxia, cough, nasal congestion, wheezing
Nair et al., 2019	Case report	n=1	16	UK	Cough, dyspnea, fever
O'Carroll et al., 2020	Case study	n=1	18	Ireland	Cough, fever, night sweats, weight loss
Odish et al., 2020	Case report	n=1	19	USA	Dyspnea
Pajak et al., 2020	Retrospective	n=3	18-20	USA	Abdominal pain, cough, diarrhea, dyspnea, nausea, vomiting
Pan et al., 2022	Case study	n=1	15	Taiwan	Abdominal pain, cough, dyspnea
Panse et al., 2020	Retrospective	n=24	17-67	USA	EVALI symptoms
Patel et al., 2020	Case study	n=1	26	USA	Abdominal pain, cough, diarrhea, nausea
Perrenoud et al., 2020	Case study	n=1	21	USA	Chill, cough, dyspnea, fever
Pitlick M., et al., 2021	Case series	n=3	20-47	USA	Chest pain, cough, dyspnea, nausea, headache
Poschenrieder et al., 2020	Case study	n=1	52	Germany	Dyspnea on exertion
Pourshahid et al., 2022	Case series	n=4	18-59	USA	Chest tightness, dyspnea, nausea
Rao et al., 2021	Retrospective	n=13	13-18	USA	General respiratory and gastrointestinal symptoms
Reddy et al., 2021	Retrospective	n=6	14-18	USA	Abdominal pain, chest pain, cough, diarrhea, dyspnea, fatigue, fever, nausea, hemoptysis, weight loss

Ring Madsen et al., 2016	Case study	n=1	45	Denmark	Abdominal pain, fever
Roman et al., 2021	Case study	n=1	31	USA	EVALI symptoms
Ronald et al., 2020	Case study	n=1	15	USA	Chills, cough, fever, vomiting
Sakla et al., 2020	Case study	n=1	25	USA	Chest pain, dyspnea, dry cough
Schekochikhina et al., 2022	Case study	n=1	35	USA	Nausea and vomiting
Silverman et al., 2020	Case series	n=3	18-21	USA	Abdominal pain, chills, dyspnea, fatigue, fever, nausea
Singh et al., 2020	Case study	n=1	20	USA	abdominal pain, fever, vomiting
Smith et al., 2020	Case study	n=1	24	USA	Nausea and vomiting
Smith et al., 2022	Case study	n=1	14	UK	Abdominal pain, chest pain, cough
Suhling et al., 2020	Case series	n=3	22-48	Germany	Cough, dyspnea, fatigue
Temas et al., 2020	Case series	n=4	19-43	USA	Cough, confusion, dyspnea, fever, nausea
Threadcraft et al., 2021	Case study	n=1	34	USA	Cough, diarrhea, dyspnea, fatigue, weight loss, nausea
Vilanilam et al., 2021	Case series	n=3	19-33	USA	Cough, diarrhea, dyspnea, fever, nausea
Viswam et al., 2018	Case study	n=1	34	UK	Lipoid pneumonia
Wang et al., 2020	Retrospective	n=11	14-18	USA	Abdominal pain, cough, dyspnea, fever, headache, nausea, vomiting, neck/back pain
Wu et al., 2020	Case study	n=1	35	USA	Dyspnea

Symbol * indicates that the study only provided the mean age

Table S2. MRI and PET report characteristics

Author	Study Type	Number of Participants	Age of Participants	Location
MRI				
Eddy et al., 2020	Case Study	n=1	18	Canada
Kizhakke Puliyakote et al., 2021	Prospective	n=9	21*	USA
Nyilas et al., 2022	Prospective	n=13	41*	Switzerland
PET				
Wall et al., 2023	Prospective	n=15	51-65	Sweden
Wetherill et al., 2023	Prospective	n=5	27*	USA

MRI= magnetic resonance imaging; PET=positron emission tomography
Symbol * indicates that the study only provided the mean age

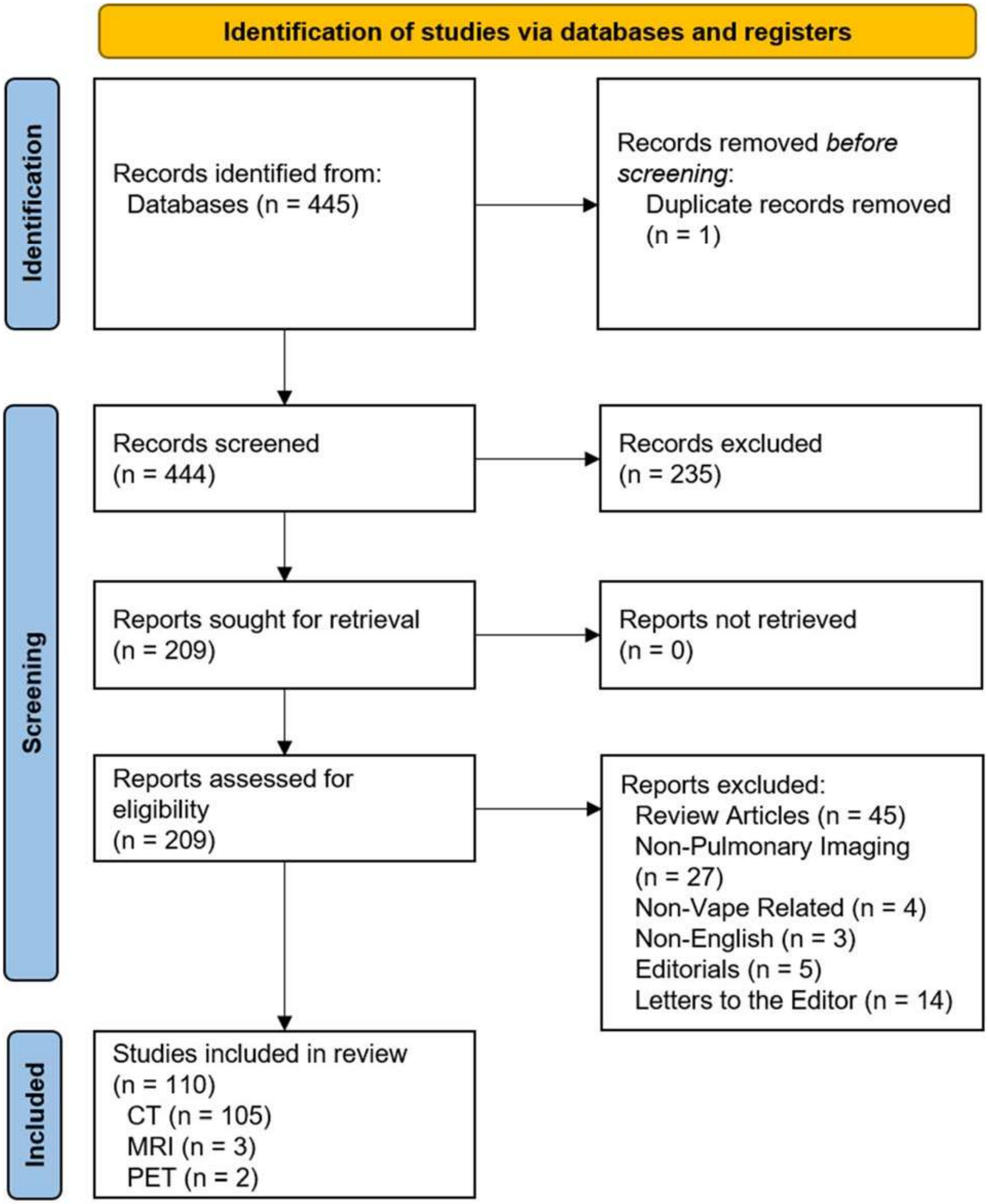

Figure S1. PRISMA flow diagram

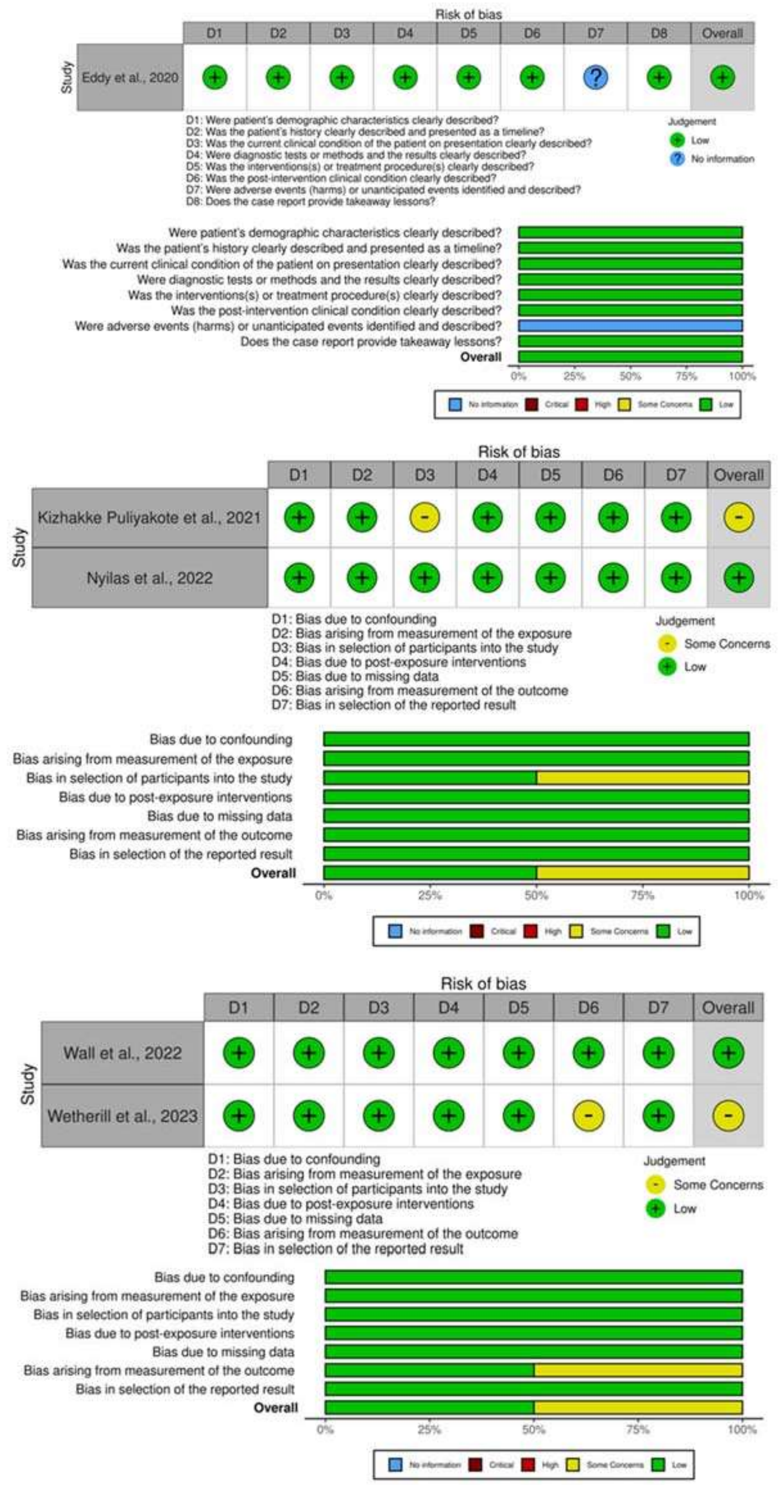

Figure S2. MRI manuscript critical appraisal checklist
